# Margination of Platelet-Sized Particles in the Red Blood Cell Suspension Flow through Square Microchannels

**DOI:** 10.3390/mi12101175

**Published:** 2021-09-29

**Authors:** Masako Sugihara-Seki, Nozomi Takinouchi

**Affiliations:** 1 Department of Pure and Applied Physics, Kansai University, Osaka 564-8680, Japan; t950019@kansai-u.ac.jp; 2 Graduate School of Engineering Science, Osaka University, Osaka 560-8531, Japan

**Keywords:** margination, near-wall excess, blood flow, platelets, cell-free layer

## Abstract

In the blood flow through microvessels, platelets show high concentrations near the vessel wall. This phenomenon is called margination of platelets and is closely associated with hemostasis and thrombosis. In the present study, we conducted in vitro experiments using platelet-sized fluorescent particles as platelet substitutes to investigate the cross-sectional distribution of these particles in the red blood cell suspension flowing through microchannels with a square cross section. Fluorescence observations were performed to measure the transverse distribution of particles at various heights from the bottom face with the use of a confocal laser scanning microscope system. In downstream cross sections of the channel, particles showed focusing near the four corners rather than uniform margination along the entire circumference of the cross section. The focusing of particles near the corners was more enhanced for higher hematocrits. On the other hand, particles in circular channel flows showed nearly axisymmetric uniform accumulation adjacent to the channel wall. The present result suggests that the segregation of suspended particles in the flow of multicomponent suspensions could have such heterogeneous 2D features of particle distribution in the cross section of channels, especially for rectangular channels often used in microfluidics.

## 1. Introduction

Platelets in the microvasculature are known to show preferential concentration near the vessel wall, which is called “margination” or “near-wall excess” [1,2]. Red blood cells (RBCs) tend to move away from the vessel wall due to the lift force generated by their deformability, so that a thin layer, called the cell-free layer (CFL), is formed in the vicinity of the vessel wall that is depleted of RBCs [3]. The internal region surrounded by the CFL is densely packed with RBCs and is called the RBC core. In microvessels, platelets are rich in the CFL and poor in the RBC core, and RBCs are the opposite. Thus, the platelet margination in the microvessel blood flow is a kind of segregation phenomenon of multicomponent suspensions observed in confined flows [4,5,6]. In fact, in vitro studies showed that the platelet margination occurs only when RBCs are present (i.e., when the volume concentration is larger than ~7%) [7]. In addition, particles with the size comparable to platelets were also reported to exhibit margination in the channel flow of the RBC suspension [7,8]. These experimental results indicate that the hydrodynamic interaction between RBCs and platelets (or particles of comparable size) causes platelet margination depending on the differences between these two types of cells, such as size, deformability, shape, and volume concentration. 

Since the margination of platelets is critical in the process of hemostasis and thrombosis, extensive studies have been performed in in vivo and in vitro experiments, numerical analyses as well as model studies. Experimental studies found that the degree of margination is affected by several factors such as the volume concentration of RBCs (hematocrit, *Ht*), channel size, flow rate, and RBC deformability [7,8,9,10,11,12]. The features of margination found in the experiments were well reproduced by numerical simulations, and it was elucidated that platelets in the RBC core are expelled toward the vessel wall by their random hydrodynamic interactions with surrounding RBCs under the influence of shear as well as volume exclusion, resulting in a high concentration of platelets in the CFL [13,14,15,16,17,18,19,20,21,22,23,24,25,26,27,28]. 

Most of these previous studies on the margination of platelets or platelet-sized particles in the RBC suspension flow have concerned circular channels [10,17,18,23,26,28] or rectangular channels with large aspect ratios [7,8,9,11]. Two-dimensional channels between two parallel plates have also been often adopted in numerical studies [15,16,19,20,21,22,24,25,27]. In these cases, only the particle distribution across the channel width was focused, and little consideration was given to its variation in the direction perpendicular to the channel width. Thus, with a few exceptions, 2D distributions of suspended particles over the entire cross section have not yet been clarified, even for rectangular channels. In a narrow rectangular channel of a 10 μm × 15 μm cross section, Zhao et al. [16] reported by a direct numerical simulation that deformed RBCs form a single train near the channel centerline and platelet-sized particles are marginated to the corners of the cross section. Quite recently, a direct numerical simulation for a 32 μm × 32 μm square channel flow done by Oh et al. [29,30] also predicted that platelet-sized particles in the RBC suspension eventually focus near the corners of the cross section. These numerical studies suggest that such inhomogeneous 2D distributions of platelets or platelet-sized particles in the cross section may be commonly expected for rectangular channel flows of the RBC suspension. Recent microfluidic studies generally adopt rectangular channels, so detailed information of the 2D distribution of suspended particles in the cross section is important in understanding the segregation characteristics of particles or cells to help in the design and optimization of microfluidic devices.

In the present study, we performed in vitro experiments to investigate the 2D distribution of platelet-sized particles in the RBC suspension flowing through square microchannels. For comparison, we also used circular channels with the same diameter to explore the 2D distribution of particles in the circular cross section. In the square channel, concentration (number density) profiles of particles across the channel width exhibited various degrees of margination depending on the height of measurement from the bottom face; they showed strong margination near the bottom face, whereas they showed weak margination near the median plane of the cross section. These variations of the concentration profile led to the 2D distribution of particles, which exhibited focusing near the four corners of the cross section rather than uniform accumulation along the entire circumference. In circular channel flows, on the other hand, particles were found to show nearly axisymmetric margination adjacent to the channel wall, as anticipated. Understanding such a highly heterogeneous 2D distribution of particles in rectangular channel flows is important for applying the segregation phenomenon of multicomponent suspensions to the separation or sorting of cells or particles. 

## 2. Materials and Methods

All of the procedures were performed according to the ethical policy of Kansai University. Fresh human blood was sampled from young, healthy volunteers and used within four hours after collection. The RBCs were washed three times by centrifugation (Model 2410 Kubota) in phosphate-buffered saline (PBS). PBS containing 1 wt% bovine serum albumin (Wako) and 5 wt% dextran (Dextran 40k Wako) was used as a suspending medium, which has a density of 1.03 × 10^3^ kg/m^3^ and viscosity of 2.5 m·Pa s at room temperature (22 °C). RBCs were suspended in this medium at *Ht* = 20% or 40%. Spherical, fluorescent polystyrene particles with a mean diameter of 2.9 μm and density of 1.05 × 10^3^ kg/m^3^ (F-XC300 Estapor) were added as platelet substitutes to the RBC suspension at the volume concentration of ~0.1%. The excitation and emission maxima of fluorescence are ~475 nm and ~525 nm, respectively.

**Figure 1:**
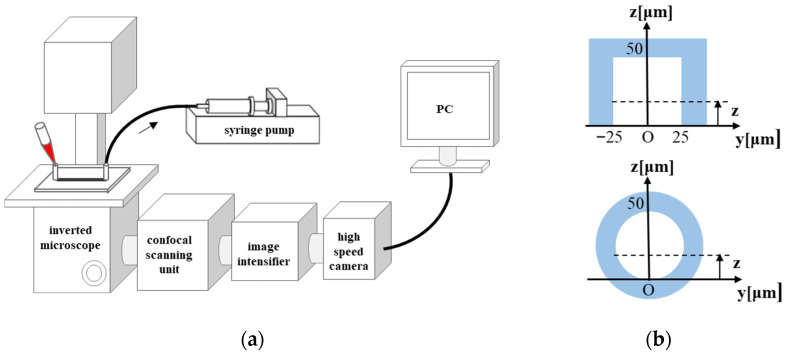
(**a**) Experimental setup; (**b**) cross sections of the square channel and circular channel. The circular channel is surrounded by water.

Figure 1 illustrates schematics of the experimental system and two types of straight flow channels with a square cross section or a circular cross section used in this study. The square channel (E3-2 Yodaka) made of polydimethylsiroxane (PDMS) has a cross section of 50 μm × 50 μm with the bottom face sealed with a glass coverslip for observation. The circular channel is a perfluoroalcoxialcan (PFA) tube with an inner diameter of 50 μm (1930 IDEX), the material of which has a refractive index of 1.34~1.35. Prior to each experiment, the channel was filled with PBS mixed with 1 wt% bovine serum albumin and left still for 1 h to coat the inner surface of the channel. The outlet of each channel was connected to a syringe, and the suspension flow was induced by a syringe pump (KDS270 KD Scientific) at constant flow rates of 1.0 μL/min for the square channel and 0.9 μL/min for the circular channel. The average flow velocities were 6.7 mmm/s and 7.6 mm/s, respectively. If we assume the flow of a Newtonian fluid, the corresponding average wall shear rates are ~950 s^−1^ and 1200 s^−1^, respectively. The maximum wall shear rate at the center of the channel face is ~1280 s^−1^ for the square channel flow. The Reynolds numbers in terms of the average flow velocity and the channel width (diameter) are 0.14 and 0.16 for the square channel flow and the circular channel flow, respectively, indicating that the inertial effect is negligible.

The fluorescent particles flowing through the microchannel were observed with a confocal laser scanning microscope system, consisting of an inverted microscope (IX71 Olympus), a confocal scanning unit (CSU-X1 Yokogawa), a laser source (Sapphire 488 Coherent), and a high-speed camera (AX50 Photron) equipped with an image intensifier (C9016-21 Hamamatsu), as shown in Figure 1a. We used a 40× oil immersion objective (UPLSAPO40XS Olympus) and a 40× water immersion objective (UAPON340 Olympus) for observing the square channel and the circular channel, respectively. The focal plane was set at various heights (*z*) from the channel bottom face up to half the height of the channel (median plane). Fluorescence observation was made at 20 mm and 50 mm downstream from the inlet (*x* = 20, 50 mm) for the square channel and the circular channel, respectively, where the margination was completed [9,11]. The images were recorded at a rate of 500 frames per second for 8 s. The pixel size of the image was 0.41 μm × 0.41 μm.

**Figure 2:**
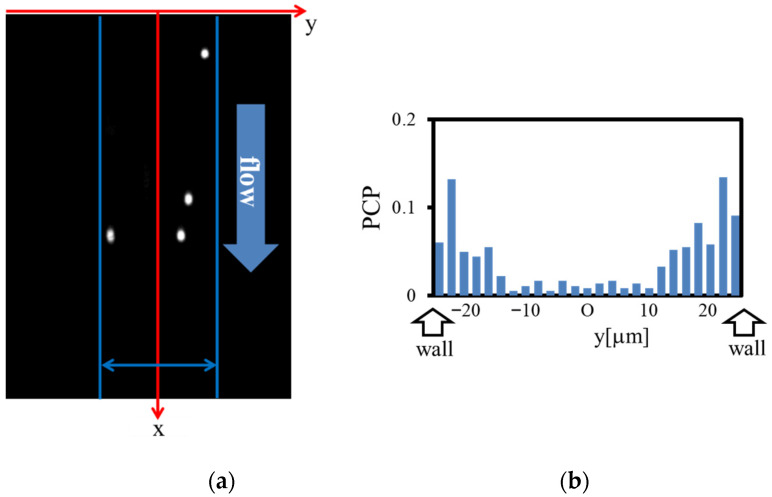
(**a**) Example of the fluorescence image obtained; (**b**) histogram representing the particle concentration profile (PCP) across the channel width.

Figure 2 shows a representative example of the fluorescence image obtained. The transverse (*y*-direction) position of the centroid of each fluorescent particle was measured with the use of an image analysis software ImageJ (NIH). We detected particles lying within about ±2 μm from the focal plane. This procedure was performed on one frame for every 50 frames of consecutive images to yield the distribution of particle numbers across the channel width. The particle concentration (number density) profile at every height (*z*) thus obtained was normalized by the total number of particles recorded and represented by a histogram (or a line graph) with its bin width of 2 μm, as shown in Figure 2b. The histograms were symmetrized with respect to the center, considering spatial symmetry. Note that the outermost particle position is limited due to the finite size of particles. However, we adopted a histogram over the entire channel width (=50 μm) for simplicity since the uncertainty of the wall position was comparable to the particle radius (~1.5 μm), as discussed in our previous study [12]. Using the particle concentration profiles in the *y*-direction for various heights *z* (≤25 μm) and the particle numbers observed, we obtained the 2D distribution of the particles over the cross section (*yz*-plane) under the assumption that the distribution is symmetric with respect to the median plane (*z* = 25 μm). The sedimentation velocity of particles was estimated to be so small (~0.04 μm/s) that the effect of particle sedimentation was neglected.

## 3. Results

**Figure 3:**
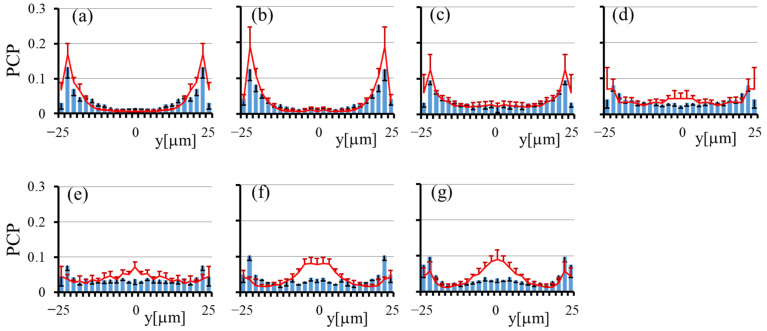
Particle concentration profiles across the channel width in the square channel: histogram for *Ht* = 20% and line graph for *Ht* = 40%, (**a**) *z* =1.9 μm, (**b**) *z* = 5.7 μm, (**c**) *z* = 9.5 μm, (**d**) *z* = 13.3 μm, (**e**) *z* = 17.1 μm, (**f**) *z* = 20.9 μm, and (**g**) *z* = 24.7 μm. The error bar represents the standard deviation (*n* = 5).

Figure 3 shows the particle concentration profiles (PCP) in the transverse (*y*) direction at *Ht* = 20% and 40% by the histogram and line graph, respectively, at various *z* for the square channel. The numbers of particles detected during the observation (5 runs) were 5772, 1602, 1114, 1138, 1141, 1149, and 1112 at *z* = 1.9, 5.7, 9.5, 13.3, 17.1, 20.9, and 24.7 μm, respectively, for *Ht* = 20%, and they were 4391, 1703, 1026, 998, 896, 828, and 748 for *Ht* = 40%. In both cases, the particle concentrations are high adjacent to the channel wall, indicating margination of the particles. The margination is especially significant at small *z*, i.e., close to the channel bottom. The peak values near the channel wall are larger for *Ht* = 40% than those for *Ht* = 20%. Near the median plane (*z*~25 μm), the concentration profiles at *Ht* = 40% attain the maximum at the center (*y*~0), suggesting accumulation of particles in the central region of the cross section. 

**Figure 4:**
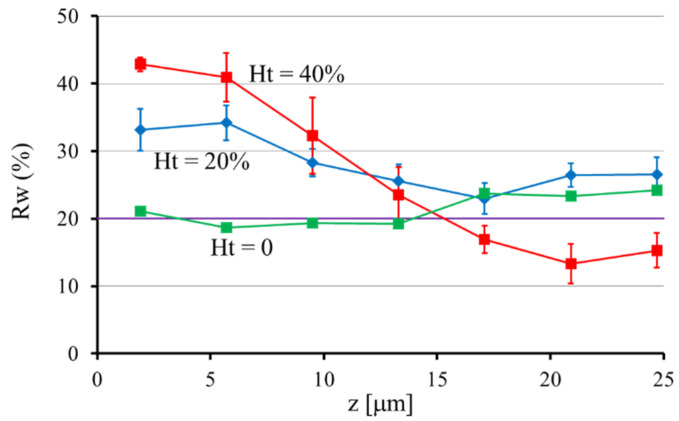
Number fraction of particles lying within 10 μm from the channel wall, *Rw*, at various heights from the bottom face of the square channel for *Ht* = 0, 20%, and 40%. For the uniform distribution of particles, *Rw* = 20%.

In order to estimate quantitatively the extent of the near-wall excess at each height, we introduced an index “*Rw*”, which is defined as the number fraction of particles lying within 10 μm from the channel wall. For the particle concentration profile obtained, we calculated *Rw* by summing the probability of five bins adjacent to the channel wall. Note that *Rw* = 20% for a uniform distribution. Figure 4 shows the variations of *Rw* with *z* at *Ht* = 20% and 40%. For comparison, the values of *Rw* at *Ht* = 0 are also plotted, which are nearly equal to 20% for all *z*. This result confirmed that particles do not marginate in the absence of RBCs. In other words, the margination of particles is caused by their interaction with surrounding RBCs. Figure 4 shows that the *Rw* values at *Ht* = 20% and 40% generally decrease with *z*, and the decrease is more significant at *Ht* = 40% than that at *Ht* = 20%. In the case of *Ht* = 40%, the *Rw* value decreases with *z* from more than 40% at small *z* to less than 20% near the median plane (*z*~25 μm). The small values of *Rw* near the median plane at *Ht* = 40% are due to the presence of the particle accumulation near the center (*y*~0), as seen in Figure 3f,g. 

**Figure 5:**
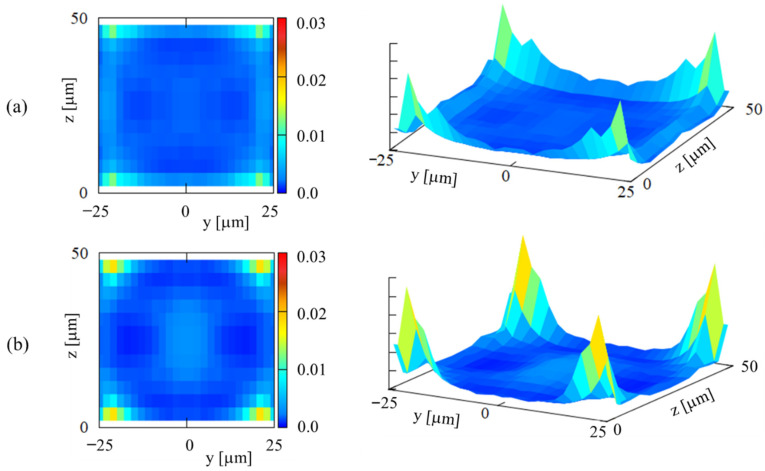
2D distributions of particles over the square cross-section at (**a**) *Ht* = 20% and (**b**) *Ht* = 40%.

Using the particle concentration profiles shown in Figure 3 and the particle numbers detected at corresponding *z*, we obtained the 2D distribution of the particles in the cross section, as shown in Figure 5. Figure 5 clearly shows that particles are focused near the four corners of the cross section rather than marginating along the entire circumference of the cross section. The particle focusing near the corners is larger at *Ht* = 40% than that at *Ht* = 20%. From the line graphs in Figure 3f,g, a significant accumulation of particles may be expected in the central region of the cross section at *Ht* = 40%. However, since the particle numbers detected near the median plane were much smaller than those near the channel bottom, only a small fraction of particles were present near the median plane, so the 2D distribution of particles has only a small bulge in the central region in Figure 5b. 

**Figure 6:**
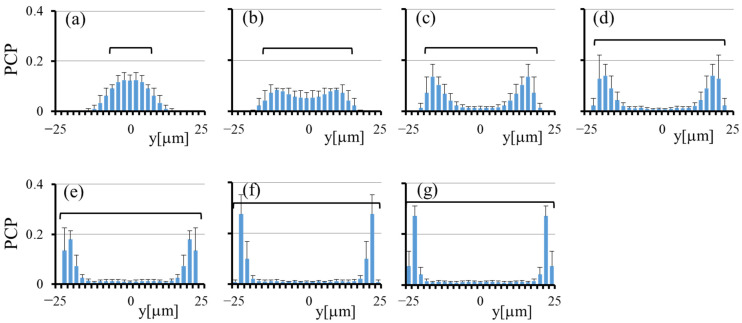
Particle concentration profiles across the channel width in the circular channel at *Ht* = 20%, (**a**) *z* =1 μm, (**b**) *z* = 5 μm, (**c**) *z* = 9 μm, (**d**) *z* = 13 μm, (**e**) *z* = 17 μm, (**f**) *z* = 21 μm, and (**g**) *z* = 25 μm. The histogram represents the average value and standard deviation (*n* = 5).

Figure 6 depicts the particle concentration profiles in the circular channel at various *z* for *Ht* = 20%. The numbers of particles detected over five runs were 3683, 6215, 5273, 5190, 4842, 4585, and 4390 at *z* = 1, 5, 9, 13, 17, 21, and 25 μm, respectively. The horizontal bar in each panel indicates the width of the circular cross section at that height. At small *z*, the particles are located around *y*~0, as expected from geometry consideration. The spread of the particle distribution wider than the width of the cross section at that height is due to a finite depth of focus in the *z*-direction. At larger *z*, the particle concentration profiles clearly exhibit near-wall excess of the particles. The histogram at the median plane shown in Figure 6g, which has high and sharp peaks close to the channel walls, is quite similar to the results obtained by previous numerical simulations for the circular channel flow [17,23,26]. 

**Figure 7:**
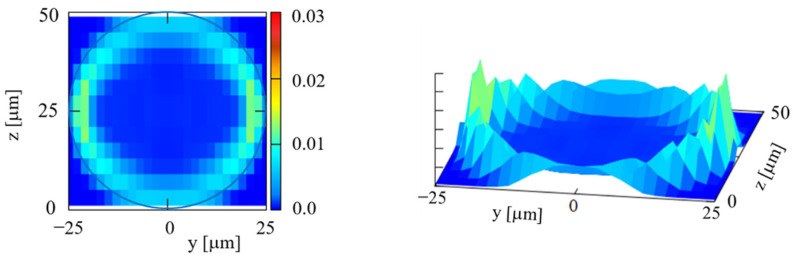
2D distribution of particles over the circular cross-section at *Ht* = 20%.

The histograms in Figure 6 were used to construct the 2D distribution of particles in the cross section, as shown in Figure 7. Figure 7 indicates a nearly uniform margination of particles along the entire circumference, as expected. This result supports the reliability of the present experimental method. Although the margination does not appear perfectly axisymmetric in Figure 7, this is mainly due to the difference in spatial resolution between the *y*- and *z*-directions, as explained in Appendix A.

## 4. Discussion

A number of numerical and model studies have been performed to elucidate the mechanism of margination of platelets in blood flow [13,14,15,16,17,18,19,20,21,22,23,24,25,26,27,28,29,30]. Crowl and Fogelson [14] conducted 2D computations to describe the development of platelet margination as a drift-diffusion process. Among more recent 3D numerical studies, Zhao and Shaqfeh [15] demonstrated that the velocity fluctuation induced by the interaction between RBCs in the RBC core causes platelets to migrate diffusively in the direction normal to the wall. Mehrabadi et al. [21] showed that platelet margination can be explained by RBC-enhanced shear-induced diffusion of platelets in the RBC core combined with platelet trapping in the CFL. Krüger [23] reported from his direct numerical simulation at *Ht* = 37% that platelets have outward averaged velocities near the edge of the RBC core, generated by the interaction with tank-treading RBCs, which facilitate the transport of platelets into the CFL. There are many other additional notable research efforts on the topic of platelet margination (see also the references of the review [31]). These studies found that the platelet margination is caused by the interaction of platelets with surrounding RBCs in the RBC core under the influence of shear, which makes platelets migrate diffusively towards the CFL. Once in the CFL, platelets rarely re-enter the RBC core. RBCs flowing near the edge of the RBC core appear to not only facilitate margination of platelets into the CFL but also act as a barrier to platelets within the CFL. Thus, platelets are trapped within the CFL, showing their excess concentration near the vessel wall.

Most of these previous studies concern the flow of circular channels, 2D channels between two parallel plates, or rectangular channels with large aspect ratios. In these configurations, the authors focused primarily on platelet migration across the channel width, with much less attention to their movement in the direction perpendicular to the width. However, Vahidkhah et al. [19] showed by their direct simulation for the motion of RBCs and platelets subject to a simple shear flow between two parallel plates that the diffusivity of platelets in the transverse direction (along the direction of the vorticity of shear flow) could also be significant due to 3D nature of the interaction between platelets and RBCs. Particularly, they pointed out the enhancement of the transverse diffusive motion of platelets within the CFL. In fact, a recent in vitro study using a bifurcating channel indicated that the transverse diffusivity of platelet-sized particles in the CFL could be comparable to previous estimates of their diffusivity in the RBC core [12]. These studies suggest the presence of significant movement of platelets or platelet-sized particles in the transverse direction in channel flows.

In in vitro experiments using a 15 μm × 10 μm rectangular channel, Hou et al. [32] measured the distribution of 3 μm spherical particles across the channel width in the RBC suspension flow. More than 90% of particles were reported to be displaced toward the channel side walls at *Ht* = 10% and 40%, but their distribution in the perpendicular direction along the side walls was unknown. However, a numerical simulation corresponding to this experiment done by Zhao et al. [16] predicted the focusing of particles near the corners of the rectangular cross section. They performed a numerical simulation of RBCs and spherical particles flowing through a 15 μm × 10 μm rectangular channel at the wall shear rate ~1000 s^−1^ and *Ht* = 10%. They reported that deformed RBCs located near the channel centerline push nearby particles toward the corners of the cross section, making them more likely to be present near the corners. Quite recently, Oh et al. [29,30] adopted the immersed boundary method and SMAC method to simulate the motion of 2.9 μm spherical particles in the RBC suspension flowing through a 32 μm × 32 μm square microchannel. Starting from nearly uniform distributions of particles, most particles were found to eventually focus near the corners of the cross section.

These results of direct numerical simulations agree with the present experimental result shown in Figure 5, which indicates the focusing of platelet-sized particles near the corners of the square cross section. In addition, Oh et al. [29,30] reported two important features. The first one is that the particles are more highly concentrated close to the channel corners at *Ht* = 40%, compared to the cases at lower *Ht*. Secondly, several particles remain near the channel center even at the final computational time at *Ht* = 40%, whereas few particles are present in the central region of the cross section at lower *Ht*. These features are also consistent with the present result of the 2D distribution of particles shown in Figure 5.

The present experimental result of the particle focusing near the corners of the cross section suggests that particles would migrate in two stages; in the first stage, they marginate in the RBC core toward the CFL, and in the second stage, they move within the CFL toward the channel corners. This migration property is confirmed by the particle trajectories reported in the numerical simulation [29,30]. In both stages, the hydrodynamic interaction of particles with RBCs (RBCs surrounding each particle in the first stage and the outermost RBCs in the second stage) mainly drives the particle movements, and the random interaction makes these movements diffusional. Although particle migration in the RBC core (the first stage) has been comprehensively studied, little is known about particle migration in the CFL (the second stage).

The diffusive motion of particles in the CFL could be attributed to their random hydrodynamic interaction with RBCs flowing at the edge of the RBC core. In a shear flow of suspension, the self-diffusion of suspended particles was intensively investigated, and a theoretical model was developed for augmented particle transport based on the shear-induced collision diffusion mechanism [33,34,35]. These pioneering studies showed that particles move diffusively due to interparticle interactions, with a diffusivity proportional to the shear rate. The linear increase in diffusivity can be intuitively understood from the increasing frequency of collision-like interaction as the shear rate increases.

**Figure 8:**
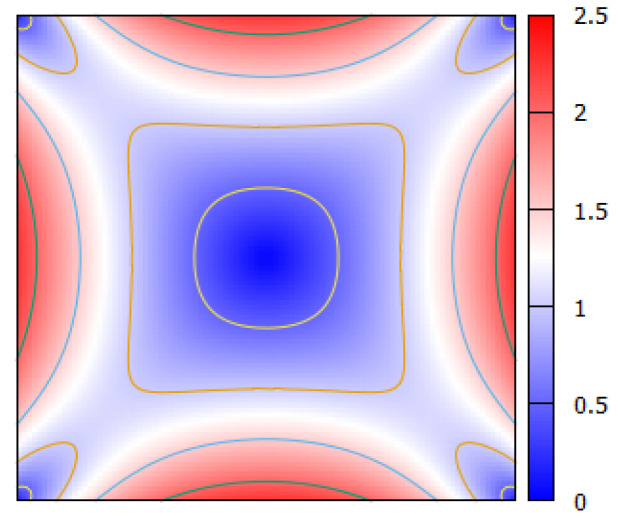
Contours of the normalized shear rate, $\left|\dot{\gamma }\right|/\left(\frac{U_{max}}{H/2}\right)$, for the flow of a Newtonian fluid in a square channel.

Similarly, in the present study, the diffusivity of particles is expected to become larger with an increasing local shear rate. Figure 8 shows the contours of the normalized shear rate, $\left|\dot{\gamma }\right|/\left(\frac{U_{max}}{H/2}\right)$, for the flow of a Newtonian fluid through a square channel, where $U_{max}$ and *H* represent the maximum velocity and the channel width, respectively. For the flow through circular channels, the normalized shear rate increases linearly with radial distance from 0 at the channel center to 2 at the wall, if *H* represents the diameter of the channel. Figure 8 shows that in square channel flows, the shear rate adjacent to the channel wall, i.e., in the CFL, varies from small values near the corners to the maximum value (>2) at the midpoint of the channel faces. This tendency suggests that, in the CFL, the diffusivity of particles is large near the midpoint of channel faces, while it is small near the channel corners. Furthermore, the direct numerical simulation by Oh et al. [29,30] demonstrated that the CFL is thicker near the corners of the square cross section compared to the rest of the circumference. From these features, it can be inferred for the second stage that marginated particles in the CFL would move diffusively along the channel wall to the corner region, and once in the corner region, they would not escape due to small fluctuations and ample space to stay.

As noted above, a recent in vitro study using rectangular channels of a 40 μm × 50 μm cross section estimated that the diffusivity of particles in the transverse direction within the CFL is similar to the diffusivity in the RBC core [12], which is consistent with the previous numerical study [19]. This estimate was done for spherical particles of 2.9 μm diameter at *Ht* = 20% and the average wall shear rates of 630–2500 s^−1^. Thus, if this result is applied to the present study, it can be inferred that the characteristic time in the second stage could be comparable to that in the first stage at *Ht* = 20%.

Figure 8 shows the presence of low shear region near the center of the cross section. At higher *Ht*, this low shear region can expand, as the non-Newtonian property of RBC suspensions is expected to flatten the velocity profile of the main flow [10,11]. Once the particles enter this low shear region, they would be trapped in this region due to small fluctuations and too small spaces between adjacent RBCs to escape easily at high *Ht*. This may account for the presence of a slight bulge near the central region in the 2D distribution of particles at *Ht* = 40% (Figure 5b).

## 5. Conclusions

In the present study, in vitro experiments were performed to investigate the 2D distribution of platelet-sized particles in the RBC suspension flowing through square microchannels. Fluorescence microscope observations to measure the transverse distribution of particles at various heights indicated that the particles show focusing near the corners of the cross section rather than uniform margination along the entire circumference of the cross section. The particle focusing near the corners was more enhanced for higher hematocrits. Understanding the presence of such a highly heterogeneous 2D distribution of particles would be important in designing microfluidic devices, which apply the segregation phenomenon to the separation or sorting of suspended particles or cells in multicomponent suspensions flowing through rectangular channels. This is especially important for dense suspensions.

## Figures and Tables

**Figure A1:**
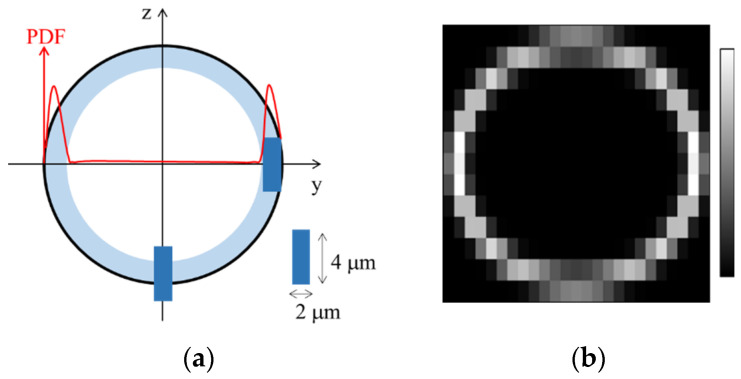
(**a**) Assumed particle concentration profile (red curve) for calculating the 2D distribution with spatial resolutions of 2 μm in the *y*-direction and 4 μm in the *z*-direction; (**b**) obtained 2D distribution of particles.

## Appendix A

In Figure 7, the particle concentration is high near the median plane, and it is low near the bottom of the circular channel. Thus, Figure 7 does not appear to show axisymmetric margination. We explain here that this is an artifact, and it is mainly due to the difference in spatial resolution between the *y*- and *z*-directions. As noted in the text, we detected particles lying within about ±2 μm from the focal plane. The particle concentration profile was drawn by a histogram with a bin width of 2 μm in the *y*-direction at every 4 μm in the *z*-direction. Correspondingly, the 2D distribution was also plotted in a grid of 2 μm and 4 μm in the *y*-and *z*-directions, respectively.

Assuming an axisymmetric concentration profile of particles, the radial profile of which was given from the experimentally obtained profile in the median plane (Figure 6g), we simulated the 2D distribution of particles with spatial resolutions of 2 μm in the *y*-direction and 4 μm in the *z*-direction. In Figure A1a, a grid with a width of 2 μm and a height of 4 μm is schematically represented by a rectangle. Near the bottom, only part of the rectangle covers the particle-rich region (light blue region), whereas near the median plane, the entire rectangle is included in the particle-rich region. Therefore, more particles are counted near the median plane than near the bottom. Figure A1b shows the simulation result for the 2D distribution. As anticipated, high concentrations are seen near the median plane, and low concentrations are widespread near the bottom, similar to Figure 7. Since Figure 7 and Figure A1b show relatively good correlation, we conclude that the particle margination shown in Figure 7 is nearly axisymmetric.

## References

[1] Tangelder G.J., Teirlinck H.C., Slaaf D.W., Reneman R.S. (1985). Distribution of blood platelets flowing in arterioles. Am. J. Physiol..

[2] Woldhuis B., Tangelder G.J., Slaaf D.W., Reneman R.S. (1992). Concentration profile of blood platelets differs in arterioles and venules. Am. J. Physiol..

[3] Kim S., Ong P.K., Yalcin O., Intaglietta M., Johnson P.C. (2009). The cell-free layer in microvascular blood flow. Biorheology.

[4] Kumar A., Graham M.D. (2011). Segregation by membrane rigidity in flowing binary suspensions of elastic capsules. Phys. Rev. E.

[5] Kumar A., Graham M.D. (2012). Mechanism of margination in confined flows of blood and other multicomponent suspensions. Phys. Rev. Lett..

[6] Kumar A., Henriquez Rivera R.G., Graham M.D. (2014). Flow-induced segregation in confined multicomponent suspensions: Effects of particle size and rigidity. J. Fluid Mech..

[7] Tilles A.W., Eckstein E.C. (1987). The near-wall excess of platelet-sized particles in blood flow: Its dependence on hematocrit and wall shear rate. Microvasc. Res..

[8] Eckstein E.C., Tilles A.W., Millero F.J. (1988). Conditions for the occurrence of large near-wall excesses of small particles during blood flow. Microvasc. Res..

[9] Fitzgibbon S., Spann A.P., Qi Q.M., Shaqfeh E.S.G. (2015). In vitro measurement of particle margination in the microchannel flow: Effect of varying hematocrit. Biophys. J..

[10] D’Apolito R., Tomaiuolo G., Taraballi F., Minardi S., Kirui D., Liu X., Cevenini A., Palomba R., Ferrari M., Salvatore F. (2015). Red blood cells affect the margination of microparticles in synthetic microcapillaries and intravital microcirculation as a function of their size and shape. J. Control. Release.

[11] Carboni E.J., Bognet B.H., Buochillon G.M., Kadilak A.L., Shor L.M., Ward M.D., Ma A.W.K. (2016). Direct tracking of particles and quantification of margination in blood flow. Biophys. J..

[12] Sugihara-Seki M., Onozawa T., Takinouchi N., Itano T., Seki J. (2020). Development of margination of platelet-sized particles in red blood cell suspensions flowing through Y-shaped bifurcating microchannels. Biorheology.

[13] AlMomani T., Udaykumar H.S., Marshall J.S., Chandran K.B. (2008). Micro-scale dynamic simulation of erythrocyte-platelet interaction in blood flow. Ann. Biomed. Eng..

[14] Crowl L., Fogelson A.L. (2011). Analysis of mechanisms for platelet near-wall excess under arterial blood flow conditions. J. Fluid Mech..

[15] Zhao H., Shaqfeh E.S.G. (2011). Shear-induced platelet margination in a microchannel. Phys. Rev. E.

[16] Zhao H., Shaqfeh E.S.G., Narsimhan V. (2012). Shear-induced particle migration and margination in a cellular suspension. Phys. Fluids.

[17] Reasor D.A., Mehrabadi M., Ku D.N., Aidun C.K. (2013). Determination of critical parameters in platelet margination. Ann. Biomed. Eng..

[18] Müller K., Fedosov D.A., Gompper G. (2014). Margination of micro- and nano-particles in blood flow and its effect on drug delivery. Sci. Rep..

[19] Vahidkhah K., Diamond S.L., Bagchi P. (2014). Platelet dynamics in three-dimensional simulation of whole blood. Biophys. J..

[20] Henriquez Rivera R.G., Sinha K., Graham M.D. (2015). Margination regimes and drainage transition in confined multicomponent suspensions. Phys. Rev. Lett..

[21] Mehrabadi M., Ku D.N., Aidun C.K. (2015). A continuum model for platelet transport in flowing blood based on direct numerical simulations of cellular blood flow. Ann. Biomech. Eng..

[22] Henriquez Rivera R.G., Zhang X., Graham M.D. (2016). Mechanistic theory of margination and flow-induced segregation in confined multicomponent suspensions: Simple shear and Poiseuille flows. Phys. Rev. Fluids.

[23] Krüger T. (2016). Effect of tube diameter and capillary number on platelet margination and near-wall dynamics. Rheol. Acta..

[24] Mehrabadi M., Ku D.N., Aidun C.K. (2016). Effects of shear rate, confinement, and particle parameters on margination in blood. Phys. Rev. E.

[25] Qi Q.M., Shaqfeh E.S.G. (2017). Theory to predict particle migration and margination in the pressure-driven channel flow of blood. Phys. Rev. Fluids.

[26] Chang H.Y., Yazdani A., Li X., Douglas K.A.A., Mantzoros C.S., Karniadakis G.E. (2018). Quantifying platelet margination in diabetic blood flow. Biophys. J..

[27] Qi Q.M., Shaqfeh E.S.G. (2018). Time-dependent particle migration and margination in the pressure-driven channel flow of blood. Phys. Rev. Fluids.

[28] Takeishi N., Imai Y., Wada S. (2019). Capture event of platelets by bolus flow of red blood cells in capillaries. J. Biomech. Sci. Eng..

[29] Oh D., Ii S., Takagi S. Numerical study of the margination of particles in red blood cells flow in a square channel. Phys. Fluids.

[30] Oh D. (2021). Numerical Simulation of the Platelet Margination Caused by the Flowing Motion of Deformable Red Blood Cells. Ph.D. Thesis.

[31] Fogelson A.L., Neeves K.B. (2015). Fluid mechanics of blood clot formation. Annu. Rev. Fluid Mech..

[32] Hou H.W., Bhagat A.A.S., Chong A.G.L., Mao P., Tan K.S.W., Han J., Lim C.T. (2010). Deformability based cell margination—A simple microfluidic design for malaria-infected erythrocyte separation. Lab Chip.

[33] Eckstein E.C., Baily D.G., Shapiro A.H. (1977). Self-diffusion of particles in shear flow of a suspension. J. Fluid Mech..

[34] Leighton D., Acrivos A. (1987). The shear-induced migration of particles in concentrated suspensions. J. Fluid Mech..

[35] Zydney A.L., Colton C.K. (1988). Augmented solute transport in the shear flow of a concentrated suspension. PCH Phys. Chem. Hydrodyn..

